# Clinical implementation of an automated VMAT treatment planning script for head and neck cancer patients: three-year experience

**DOI:** 10.3389/fonc.2026.1877137

**Published:** 2026-07-03

**Authors:** Nataliya Kovalchuk, Peng Dong, Caressa Hui, Ignacio Romero, Ziyi Wang, Lina Shah, Raveena Pandya, Michael Xiang, Everett J. Moding, Michael F. Gensheimer, Beth M. Beadle, Quynh-Thu Le, Lei Xing, Yong Yang

**Affiliations:** 1 Department of Radiation Oncology, Stanford University, Stanford, CA, United States; 2 Department of Radiation Oncology, University of California, Irvine, Irvine, CA, United States; 3 Department of Radiation Oncology, Stanford Health Care, Stanford, CA, United States

**Keywords:** automation, autoplanning, head and neck cancer, radiation therapy, treatment planning

## Abstract

**Purpose:**

To assess the impact of implementing an in-house automated volumetric modulated arc therapy (VMAT) planning script for patients with head and neck (HN) cancer.

**Methods:**

The automated planning script was implemented at our institution in April 2020. During validation, 10 auto-plans were compared with 10 corresponding manual plans for dosimetric indices, Five radiation oncologists blindly reviewed these plans for clinical acceptability and treatment preference. For clinical evaluation, dosimetric indices from 1000 HN patients consecutively treated between 2017 and 2023 (500 manual pre-implementation, 500 automated post-implementation) were compared using t-tests (p<0.05).

**Results:**

In validation testing, 10 auto-plans maintained PTV D_95%_ prescription coverage and similar global D_max_ to corresponding manual plans (107.4% vs 107.3%) while significantly reducing maximum doses D_max_ to the brainstem (-5.1 Gy) and spinal cord (-2.9 Gy), both p < 0.03, and mean doses to the ipsilateral parotid (-4.8 Gy), esophagus (-3.9 Gy), cochleae (-3.8 Gy), contralateral submandibular gland (-3.6 Gy), contralateral parotid (-2.2 Gy), and pharynx (-2.0 Gy), all p < 0.05. In blinded review by physicians, 94% of automated plans and 86% of manual plans were rated clinically acceptable and most physicians preferred 7 auto-plans.

In the clinical evaluation phase following three years of clinical use, 500 auto-plans achieved PTV D_95%_ prescription coverage and similar global D_max_ to manual plans while significantly reducing maximum doses to the brainstem (-3.6 Gy) and spinal cord (-2.1 Gy), both p < 0.001. In addition, auto-plans demonstrated significant mean dose reductions for the contralateral submandibular gland (-4.1 Gy), ipsilateral parotid (-3.9 Gy), oral cavity (-2.5 Gy), cochleae (-2.4 Gy), larynx (-2.0 Gy), contralateral parotid (-1.5 Gy), and maximum doses to the mandible (-2.9 Gy) and lips (-2.3 Gy), all p < 0.04.

**Conclusions:**

Automated planning improved organ-at-risk sparing without compromising target coverage or dose homogeneity, with high clinical acceptability.

## Introduction

Radiotherapy for head and neck (HN) cancers presents a unique challenge due to the complex anatomy, close proximity of multiple critical structures, and the need for high-dose coverage of irregular target volumes. Intensity-Modulated Radiation Therapy (IMRT) and Volumetric Modulated Arc Therapy (VMAT) become a standard technique in HN cancer treatment, offering improved dose conformity and sparing of organs at risk (OARs), significantly enhancing patient outcomes and quality of life (1–5). However, VMAT planning for HN cancer remains one of the most time-consuming and expertise-dependent tasks in clinical radiation oncology. Manual plan generation requires extensive contour review, iterative optimization, and fine-tuning to meet target coverage and OAR constraints, which can lead to variability in plan quality between planners and institutions (6).

Automation in treatment planning has emerged as a promising strategy to address these limitations. Advances in knowledge-based planning, template-driven optimization, and scripting through platforms such as the Eclipse Scripting Application Programming Interface (ESAPI) have enabled the development of automated workflows capable of producing high-quality plans with minimal user intervention (7). These automated planning systems have the potential to improve planning efficiency, reduce inter-planner variability, and enhance plan quality, while freeing clinical staff to focus on other patient care activities.

Although automated planning has been evaluated in various disease sites (8, 9), there is limited large-scale clinical evidence assessing its long-term impact on plan quality and clinical acceptance in HN cancer. Most published studies are limited to small retrospective cohorts or feasibility testing, with little data on multi-year, real-world use (10, 11). Furthermore, while prior work suggests automated planning can achieve dosimetric quality comparable or superior to manual planning, the magnitude and consistency of organ sparing benefits across large patient populations remain insufficiently characterized (12).

To address these gaps, we implemented an in-house automated VMAT planning script for patients with HN cancer in April 2020 and conducted a two-phase evaluation: (1) a validation phase comparing automated and manual plans for a sample of previously treated patients, including blinded physician review; and (2) a clinical evaluation phase assessing dosimetric outcomes in 1000 consecutive patients before and after introducing automation. This study aims to provide robust evidence of the clinical impact, and acceptance of automated VMAT planning in HN cancer.

## Methods

### Autoplanning script development

The methodology behind the dose prediction model used in autoplanning is based on Ma et al. using deep convolution neural network and predicting the dose from anatomic features and PTV-only plans dosimetric features (13).

**Figure 1:**
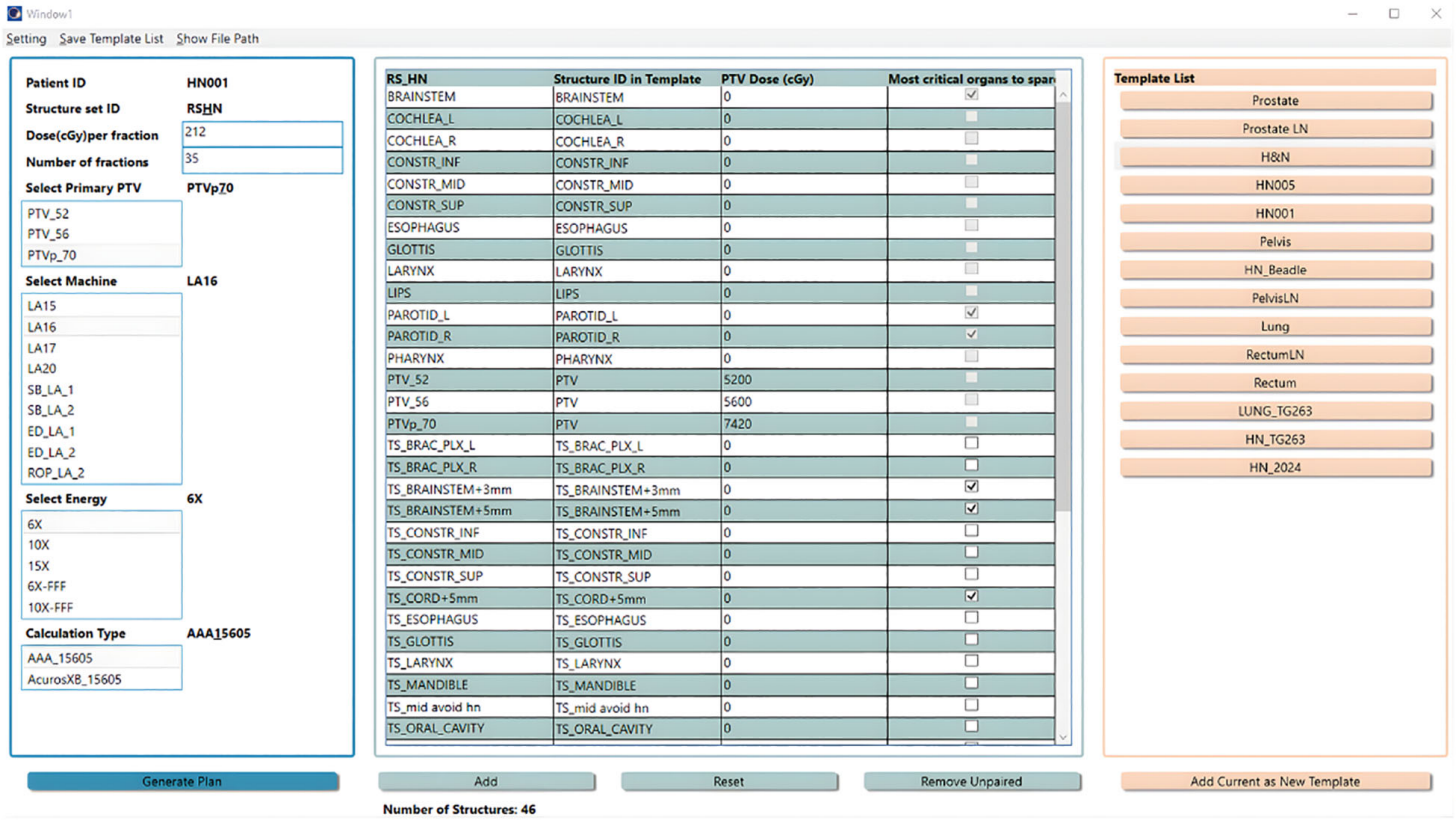
Shows the graphical user interface (GUI) for the auto-planning script.

The application programming interface (API) script was developed within version 15.6 of the Varian Eclipse Scripting API (ESAPI) to facilitate HN auto-planning for radiation therapy (**Figure 1**). The auto-planning process was divided into two key stages: preparation and optimization. By dividing the workflow, planners have an opportunity to review the generated optimization structures, the created plans, isocenter and beam placements, and assigned optimization objectives before proceeding with the optimization loop. This allows for corrections to be made early, avoiding unnecessary time spent optimizing suboptimal setups.

Once the organs at risk structures and targets are approved by the physician, the planner initiates the preparation part of the script. The script generates optimization structures, sets up beams and initializes optimization parameters. After completing the plan preparation, the user saves the work, reloads the patient data in Eclipse, and reviews the script output.

When satisfied with the prepared plan, the user launches the optimization loop script either within Eclipse or outside the Eclipse on a dedicated workstation. The script continues where the preparation left off, starting from running a PTV-only plan to provide a patient-specific plan objectives and optimization constraints. Once the optimization loop begins, it runs until all objectives are met or a predefined number of iterations is reached. Optimization parameters are automatically updated based on plan evaluator, hot and cold spots are autogenerated to improve plan homogeneity. A few optimization iterations are run until all constraints are met, or the preset maximum iteration number is reached.

### Validation phase

The developed script was tested on a cohort of 10 patients previously treated at our institution before its clinical launch in 2020. The 10 validation cases represented a range of disease sites and were selected to reflect the diversity of patients treated clinically at our institution (oropharynx, oral cavity, hypopharynx, and nasopharynx cancer cases). Dose calculations for both manual and automated treatment plans were performed using the Acuros XB dose calculation algorithm in Eclipse (Varian Medical Systems, Palo Alto, CA). The quality of the auto-generated plans was compared to their manually created clinical counterparts using clinically relevant dose-volume histogram (DVH) metrics for organs at risk and targets. Paired t-tests were performed to assess the significance of observed differences, with statistical significance set at p ≤ 0.05.

To further assess plan quality, a retrospective blinded review was conducted by five physicians, where all identifying information was removed from the 20 plans under evaluation. Reviewers were asked two key questions for each patient: (1) whether each plan was clinically acceptable, and (2) which of the two plans, manual or auto-generated, they would choose for treatment and the reasons for their choice.

### Clinical implementation phase

Three years after auto-planning script implementation, another ESAPI script was developed to query Eclipse database to extract the auto-plans sequentially post-clinical implementation. The dosimetric indices for 500 manual plans consecutively treated between January 2017 and March 2020 were compared with 500 auto-plans consecutively generated between April 2020 and March 2023. Patients were treated using standard institutional head and neck radiotherapy prescriptions, including 69.96 Gy in 33 fractions, 70 Gy in 35 fractions, 66 Gy in 30 fractions, 66 Gy in 33 fractions, 60 Gy in 30 fractions, and other risk-adapted dose levels delivered using a simultaneous integrated boost technique. Continuous variables were compared using student’s t-test, and categorical variables were compared using the chi-square test, with p < 0.05 indicating statistical significance.

## Results

### Autoplanning script development

The in-house HN autoplanning script (**Table 1**) was successfully implemented within Eclipse v15.6 with a two-stage automated workflow. The preparation stage consistently generated complete plan setups, including isocenter placement, beam geometry, dose calculation setup, optimization structures, and optimization parameters under 5 minutes. This stage allowed planners to review and, when necessary, modify optimization structures, beam placements, and objectives before proceeding to full optimization.

**Table 1:** Patient, disease, and treatment characteristics of head and neck cancer treatments before and after the auto-planning script during the clinical implementation phase.

Patient characteristics	Before auto-planning, N (%)	After auto-planning, N (%)	P value
Age at RT (mean ± SD, years)	69.4 ± 13.4	67.3 ± 13.4	0.01*
Gender			<0.01*
Male	384 (76.8%)	342 (68.4%)	
Female	116 (23.2%)	158 (31.6%)	
RT Dose (mean ± SD, Gy)	64.3 ± 6.8	63.6 ± 6.5	0.13
Planning Target Volume (mean ± SD, cc)	583.2 ± 250.1	559.8 ± 255.5	0.61
Treatment Laterality			0.98
Unilateral	112 (22.4%)	115 (23.0%)	
Bilateral	388 (77.6%)	385 (77.0%)	
MU (mean ± SD)	642.8 ± 197.0	667.2 ± 186.3	0.07
Primary subsite			0.12
Oral Cavity	78 (15.6%)	102 (20.4%)	
Oropharynx	177 (35.4%)	141 (28.2%)	
Hypopharynx	20 (4.0%)	18 (3.6%)	
Nasopharynx	28 (5.6%)	21 (4.2%)	
Larynx	30 (6.0%)	30 (6.0%)	
Major Salivary Glands	38 (7.6%)	57 (11.4%)	
Nasal Cavity & Paranasal Sinuses	42 (8.4%)	38 (7.6%)	
Thyroid	12 (2.4%)	11 (2.2%)	
Other/Head & Neck NOS	75 (15.0%)	82 (16.4%)	

Statistically significant differences (p < 0.05) are bolded and marked with an asterisk.

Following planner review, the optimization stage produced final plans in 50–60 minutes on standard clinical workstations. With the Graphics Processing Unit (GPU) used in optimization, the optimization time can be reduced to 15–20 minutes. Maximum five optimization loops automatically adjusted objectives, generated hot and cold spot structures, and iteratively refined dose distribution until convergence criteria were met. Across validation cases, the optimization stage consistently achieved PTV coverage targets and met institutional organ-at-risk constraints without requiring manual re-optimization.

The two-stage approach proved robust across a wide range of patient anatomies and target complexities. In both validation and subsequent clinical use, the script reproducibly delivered high-quality plans with minimal manual intervention. User feedback indicated that the ability to pause between preparation and optimization was valuable for quality assurance, particularly in cases with unusual anatomy or challenging target/OAR relationships. .

### Validation phase

**Figure 2:**
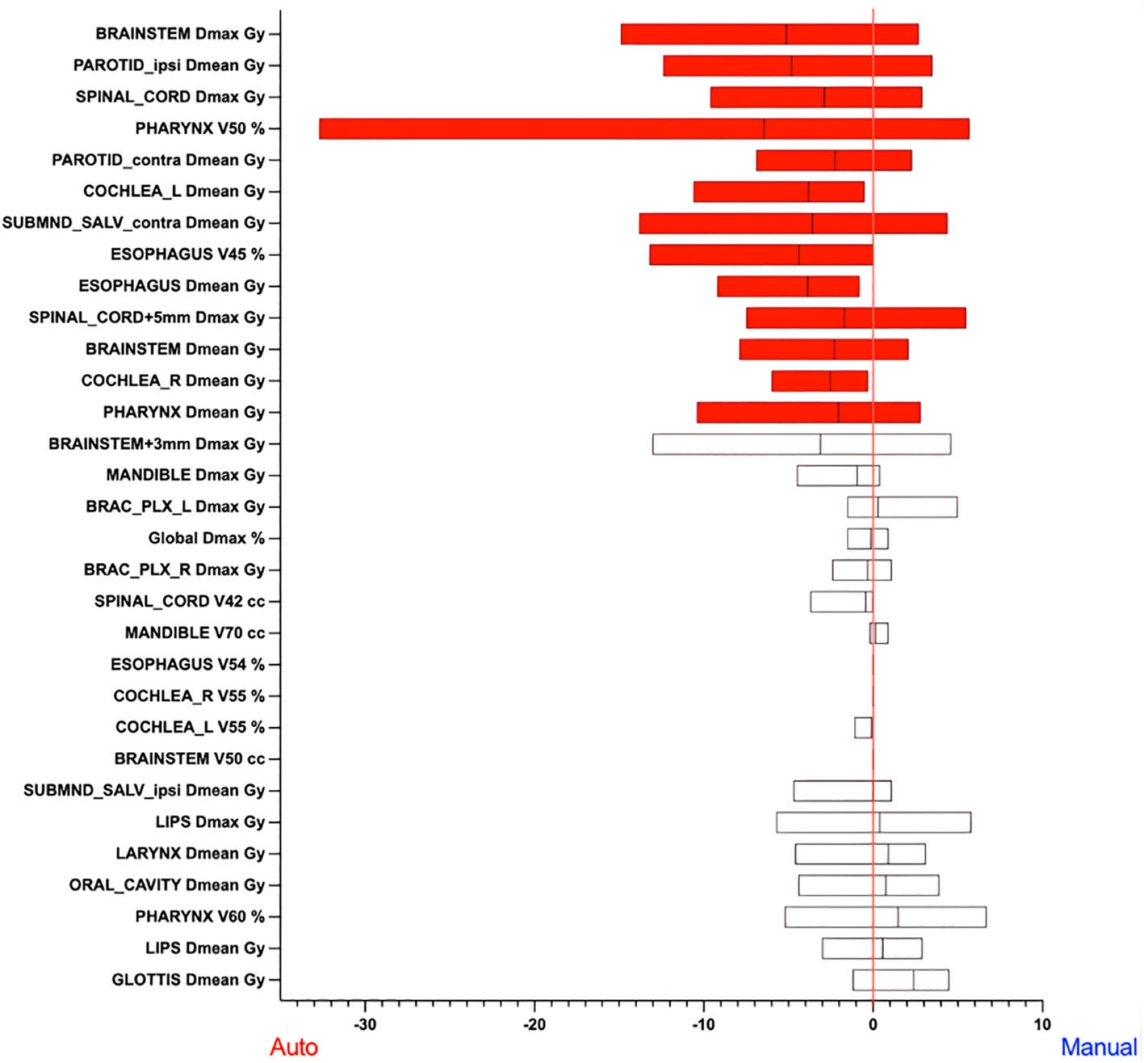
Box plots (middle line at mean and extending to range) show the differences in achieved plan quality metrics between the auto-plans and manual plans for a validation cohort of 10 patients with head and neck cancer. Parameters plotted in the left part of the graph show auto-plan superiority. Red shading denotes statistically significant differences. The differences in plan dose heterogeneity were not statistically significant.

**Figure 3:**
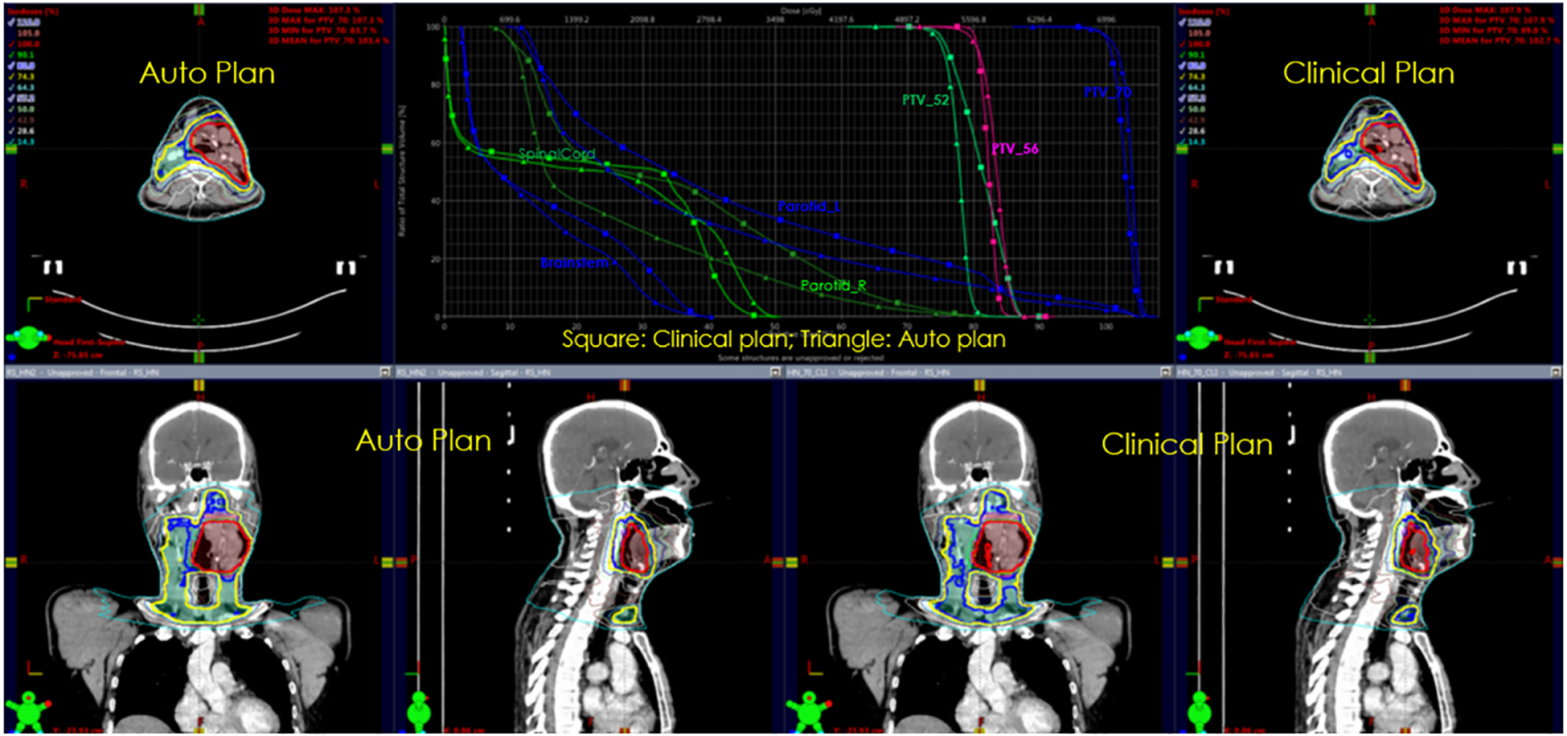
Isodose comparison between auto- and manual plan for one of the validation patients.

For the 10 HN plans used for validation, compared to the corresponding manually generated clinical plans, there was no statistically significant change in average global D_max_ (107.4% vs 107.3%). All auto-plans achieved PTV D_95%_ coverage and reduced maximum doses to brainstem and spinal cord with mean ± standard deviation reductions of 5.1 ± 2.6 Gy and 2.9 ± 1.4 Gy, respectively (all p <0.03). Auto-plans also reduced mean doses to the contralateral parotid, ipsilateral parotid, contralateral submandibular gland, pharynx, esophagus, and cochlea, with mean ± standard deviation reductions of 2.2 ± 2.9 Gy, 4.8 ± 4.7 Gy, 3.6  ± 5.2 Gy, 2.0 ± 7.1 Gy, 3.9 ± 2.6 Gy, 3.8 ± 5.0 Gy respectively (all p < 0.045). **Figure 2** shows the box plots of differences in achieved plan quality between auto-plans and manual plans for the validation cohort of 10 patients with head and neck cancer. **Figure 3** shows the isodose and DVH comparisons between auto- and manual plan for one of the patients from the validation cohort. Compared with manual planning, the automated plan achieved similar target coverage and dose homogeneity, while providing improved sparing of most organs at risk.

**Figure 4:**
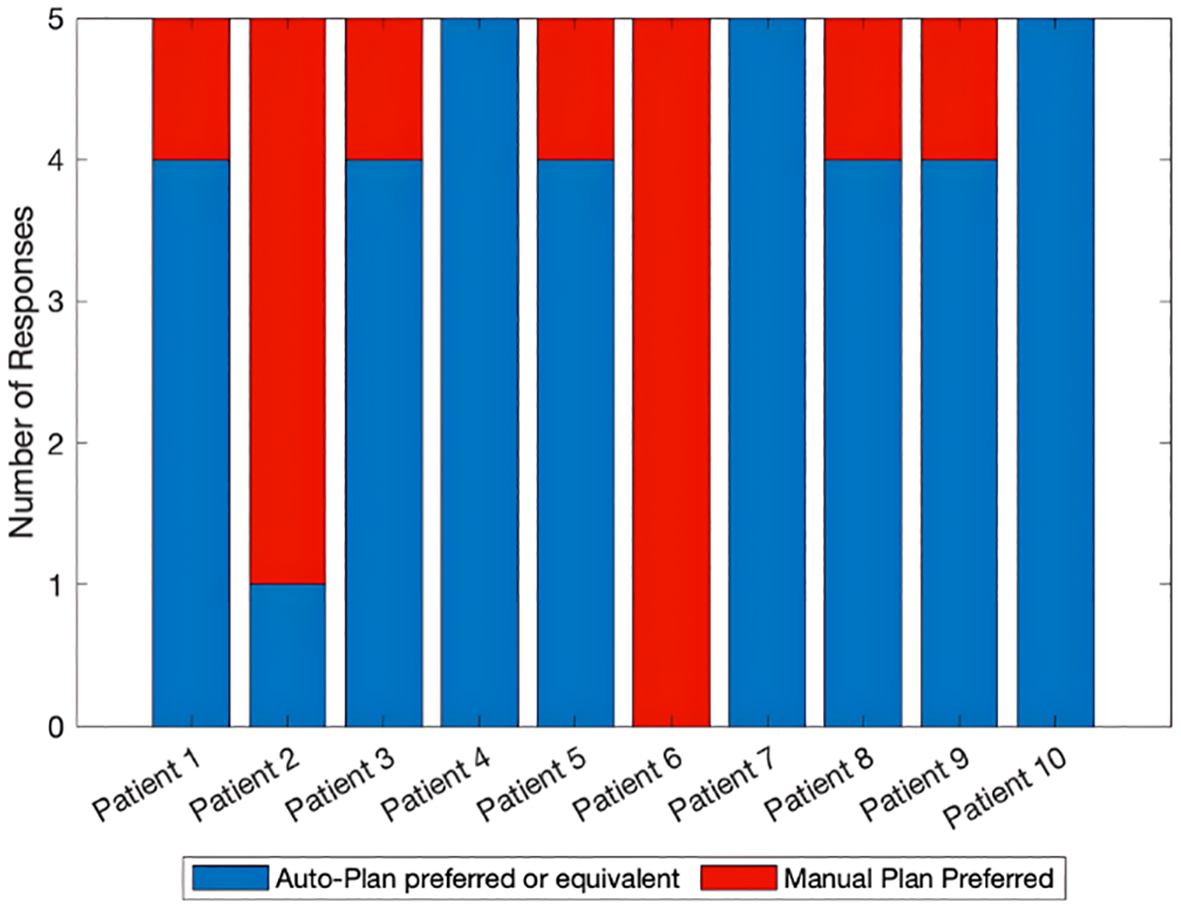
The blinded plan review results by 5 physician experts: blue denotes auto-plan preference or equivalency to manual plan, and red shows manual plan preference.

In addition, in the blinded review by 5 physicians, out of 50 responses 94% considered auto-plans clinically acceptable versus 86% for manual plans. Overall, 7 auto-plans were preferred for treatment by the majority, 1 was deemed equivalent, while only 2 manual plans were preferred (**Figure 4**).

### Clinical implementation phase

In the clinical evaluation phase, 1000 HN treatment plans were analyzed, including 500 consecutively treated manual plans before the auto-planning script was clinically implemented (January 2017 - March 2020), and 500 consecutive plans after its clinical implementation (April 2020 - March 2023).

**Table 1** shows the patient, disease, and treatment characteristics for 2 cohorts: plans before and after the auto-planning script. This data indicates that after auto-planning cohort had a slightly lower mean age (67.3 vs 69.4 years, p = 0.01), and a higher proportion of female patients (31.6% vs 23.2%, p < 0.01). Other characteristics, including prescribed RT dose, treatment laterality, average planning target volumes and monitor units, were similar between groups.

Compared to 500 previously treated manually generated clinical head and neck plans, the 500 auto-plans achieved PTV D_95%_ coverage and critical organs at risk sparing without statistically significant change in average global D_max_ (107.4% for manual vs 107.5% for auto-plans). The auto-planning solution provided statistically significant reductions in maximum doses to brainstem and spinal cord (average reductions of 3.6 ± 0.1 Gy and 2.1 ± 1.1 Gy, respectively, (all p <0.001), significantly reduced average mean doses to contralateral submandibular gland, ipsilateral parotid, oral cavity, cochleae, larynx, contralateral parotid (reductions of 4.1 ± 1.2 Gy, 3.9 ± 0.4 Gy, 2.5 ± 0.1 Gy, 2.4 ± 0.2 Gy, 2.0 ± 1.4 Gy, 1.5 ± 0.1 Gy, respectively, all p < 0.03) and significantly reduced average maximum doses to mandible and lips (reductions of 2.9 ± 2.8 Gy and 2.3 ± 1.2 Gy, respectively, all p < 0.04).

**Table 2** summarizes the average dosimetric indices and their percent differences between manual and auto-plan cohorts of the clinical implementation phase. Auto-planning significantly reduced doses to multiple organs at risk, with the largest average reductions observed for the mandible V70 Gy (-57.7%), right cochlea mean dose (-17.0%), left cochlea mean dose (-15.9%), brainstem Dmax (-12.0%), and ipsilateral parotid mean dose (-11.6%) (all p < 0.05). No clinically meaningful differences were observed in global Dmax or doses to structures such as the brachial plexus and esophagus.

**Table 2:** Dosimetric comparison of 500 manually planned and 500 auto-planned head and neck cancer treatments during the clinical implementation phase.

	Manual		Auto		% Diff (Auto-manual)	
DVH Metric	Ave	SD	Ave	SD	Ave	P value
MANDIBLE V70Gy, cc	1.7	4.2	0.7	2.4	-57.7%	<0.001*
COCHLEA_R Dmean, Gy	15.1	13.3	12.5	13.4	-17.0%	0.004*
COCHLEA_L Dmean, Gy	14.3	13.0	12.0	12.5	-15.9%	0.007*
BRAINSTEM Dmean, Gy	10.8	7.5	9.2	7.4	-15.4%	<0.001*
BRAINSTEM+3mm Dmax, Gy	35.2	13.5	30.7	14.1	-12.8%	<0.001*
BRAINSTEM Dmax, Gy	29.8	12.5	26.2	12.6	-12.0%	<0.001*
PAROTID_IPSI Dmean, Gy	34.0	17.3	30.0	16.9	-11.6%	<0.001*
SUBMND_SALV_CONTRA Dmean, Gy	38.9	21.5	34.7	22.7	-10.7%	0.008*
SPINAL_CORD+5mm Dmax, Gy	38.9	10.5	35.1	10.6	-9.8%	<0.001*
ORAL_CAVITY Dmean, Gy	31.5	13.8	29.0	13.9	-8.0%	0.005*
PAROTID_CONTRA Dmean, Gy	19.6	10.5	18.2	10.5	-7.4%	0.024*
SPINAL_CORD Dmax, Gy	30.8	10.2	28.7	9.2	-6.8%	<0.001*
LIPS Dmax, Gy	36.0	15.8	33.7	17.0	-6.3%	0.041*
LARYNX Dmean, Gy	32.2	13.5	30.3	14.9	-6.1%	0.026*
MANDIBLE Dmax, Gy	65.3	11.2	62.4	14.0	-4.5%	<0.001*
GLOTTIS Dmax, Gy	45.3	17.3	43.4	18.2	-4.3%	0.060
PHARYNX V50Gy, %	33.5	26.5	29.8	26.4	-3.7%	0.025*
PHARYNX Dmean, Gy	37.7	15.0	36.7	14.4	-2.7%	0.175
SUBMND_SALV_IPSI, Dmean, Gy	51.2	22.9	50.3	21.0	-1.8%	0.275
PHARYNX V60Gy, %	17.9	21.2	16.2	22.1	-1.7%	0.145
ESOPHAGUS V45Gy, %	7.0	15.0	5.4	13.5	-1.6%	0.051
BRAC_PLX_L Dmax, Gy	51.6	17.9	51.0	18.2	-1.1%	0.335
COCHLEA_L V55Gy, %	2.2	14.0	1.7	12.8	-0.5%	0.319
ESOPHAGUS V54Gy, %	3.0	12.1	2.8	11.0	-0.2%	0.408
COCHLEA_R V55Gy, %	2.4	14.4	2.5	15.4	0.1%	0.451
BODY Dmax, %	107.4	1.6	107.5	1.3	0.1%	0.057
LIPS Dmean, Gy	18.7	10.0	18.9	11.4	1.0%	0.412
BRAINSTEM V55Gy, cc	0.0	0.0	0.0	0.1	1.3%	0.495
BRAC_PLX_R Dmax, Gy	51.4	17.7	52.2	17.3	1.5%	0.283
ESOPHAGUS Dmean, Gy	15.9	10.5	16.7	10.0	5.2%	0.121
**Average**	-7.0%					

Values represent average (Ave) and standard deviation (SD) for each dose–volume histogram (DVH) metric. Negative values of percent difference indicate reduced dose with auto-planning. Statistically significant improvements (p < 0.05) are marked with bolding and asterisk.

## Discussion

This single institution study is the largest series comparing the efficacy of automated radiation treatment planning to manual radiation treatment planning in definitively treated head and neck cancer cases. In this cohort of patients, automated treatment planning resulted in improved plan quality with significantly decreased doses to multiple organs at risk compared to the manually planned treatments without sacrificing target coverage and dose homogeneity. Both the validation phase and the multi-year clinical implementation phase confirmed consistent dosimetric benefits across multiple critical structures, reinforcing the robustness and reproducibility of automated planning in a challenging disease site. The results of this study have potential widespread implications on access to advanced head and neck treatment planning, which historically has been a resource-intensive and user expertise-dependent process.

Our validation cohort showed that auto-plans reduced maximum doses to the brainstem and spinal cord and lowered mean doses to multiple salivary glands, oral cavity, and cochleae, all while maintaining prescription coverage. These improvements were corroborated by physician review, in which auto-plans were not only deemed clinically acceptable in the majority of cases, but actually preferred over manual plans in most comparisons. Importantly, these findings translated to the clinical implementation phase, where analysis of 1000 consecutive patients revealed statistically significant dose reductions across nearly all major OARs. Collectively, these results provide strong evidence that automation can achieve consistent dosimetric advantages in routine clinical use.

Although numerous machine learning models and algorithms have shown significant promise for clinical use in head and neck treatment planning, most are currently tested in simulated environments that do not fully mirror real-world clinical workflow (12, 14, 15). A study by McIntosh et al. compared machine learning and human generated RT treatment plans in a retrospective simulation with retesting and a prospective clinical deployment phase found that although 83% of the radiation therapy plans generated during the simulation phase were selected for treatment, only 61% were chosen once the models were deployed in the clinical setting. These results highlight the gap between simulation and practical implementation and emphasize the need for judicious translation of machine learning methods into clinical care. Some common barriers to the clinical adaptation of machine learning in medicine noted by McIntosh et al. include performance reliability and applicability, safety, integration into workflow, and trust (14). Head and neck VMAT in particular requires a highly complex planning process due to not only numerous adjacent organs at risk and but also often several prescription volumes necessitating highly computational algorithms (16). Thus, there is a paucity of literature on the feasibility of deep learning algorithms in head and neck radiotherapy planning, and data on the implementation of these techniques in real-world clinical environments is further limited.

Our results from this study compare favorably to an automated virtual integrative knowledge-based planning application for head and neck cancer plans described by Jaworski et al (17). This particular script was developed from 668 previously treated head and neck RT plans and retrospectively validated in a cohort of 52 head and neck cases where 86% of plans were safe to treat, with 10% minor and 4% major revisions or rejection recommended. Notably, this study highlighted the importance of disease subsite-specific performance of the automated virtual integrative-planner algorithm and found that while all oropharynx, p16-positive squamous cell cancer of unknown primary, larynx, and hypopharynx plans were “treat as is”, major revisions or rejections were more often recommended for sinonasal, nasopharynx, p16-negative squamous cell carcinoma of unknown primary, and cutaneous subsites. The authors also reported that minor revisions comprised of increasing conformality and reducing heterogeneity, while major revisions were limiting hot spots outside PTV, restricting hotspots within the PTV to 105% to 110%, and improving target coverage (17). Overall, the percentage of clinically acceptable plans were similar when compared with our study.

In another blinded review by specialized head and neck physicians, 94% of auto-plans were deemed clinically acceptable as is without any edits in our study, which compares favorably to the 88% by Radiation Planning Assistant (RPA) (18). In this blinded study of a cohort of 50 patients with head and neck cancer, 14 specialized head and neck radiation oncologists were instructed to review the clinical acceptability of an auto-plan, which was created with the RPA, and a clinical plan, which was manually created with interaction between the treatment planner and the physician, approved, and used for clinical treatment. The reviewers scored 78% of the clinical plans and 88% of the auto-plans as being usable, and when asked to choose which play they would prefer, reviewers chose the auto-plan or either for 72% of the plans and the clinical plan or either for 52% of the plans (19). The current RPA system hardware takes approximately 30 minutes to complete a VMAT head and neck plan, which includes just one optimization; this is in contrast to our auto-planning script that runs up to five optimizations. Together, these findings underscore that automated planning not only delivers high-quality head and neck treatment plans with superior clinical acceptability but also achieves this with greater efficiency through multiple optimization cycles, highlighting its potential to streamline workflows.

Compared to the manually generated clinical head and neck plans by experienced planners, the auto-plans in our study significantly reduced doses to adjacent critical organs at risk and were more likely to be deemed clinically acceptable by physicians. These results are consistent with the experience published by Gao et al, where a virtual treatment planner was found to outperform the corresponding clinical plans generated manually in 20 comparative plans, with superior dose sparing of organs at risk, including the brainstem, spinal cord, parotid glands, oral cavity, esophagus, and pharyngeal constrictor, without sacrificing target volume coverage. However, unlike our study, the plans generated by this virtual treatment planner had significantly higher MUs, with an average of 795.35 ± 125.18 MU per fraction, compared to human-generated plans with 607.43 ± 108.16 MU. Our results showed a similar trend, with auto-plans exhibiting slightly higher MUs, suggesting greater intensity modulation; however, these differences did not reach statistical significance. In Gao et al. work, the plans were produced in an average of 91 minutes, compared to 50–60 minutes for our automatically generated plans (20). However, in contrast to our results where dose homogeneity was not sacrificed, a small retrospective study evaluating an AI tool where training, validation, and testing datasets consisting of 200, 16, and 15 retrospective cases reported worse dose heterogeneity and global maximum dose (21). These findings highlight that our in-house automated planning approach achieves consistent OAR sparing and high clinical acceptability without compromising dose homogeneity, distinguishing it from other reported automated and AI-based planning solutions.

There are several limitations to our study and current auto-planning system. Although objective dosimetric parameters are significantly improved compared to manual plans, whether these reduced doses to organs at risk translate into clinical benefit with decreased patient toxicities is unknown, as toxicity data was not collected. However, our study aims to provide reasonable alternatives for time-consuming human-centered treatment planning to mitigate a historically laborious workflow. Further studies with focus on clinical outcomes will need to be performed to ascertain the clinical benefit or equivalence of auto-plans to manual plans.

In routine clinical practice, auto-plans generated by the script were reviewed by dosimetrists and physicians prior to final approval, and minor manual adjustments might have been performed to address patient-specific considerations or institutional preferences. These clinically finalized plans, including those with limited dosimetrist refinements, were included in the post-implementation cohort, reflecting real-world use of the auto-planning script. As such, the reported results represent the performance of the automated workflow within standard clinical quality assurance processes rather than a purely “hands-off” planning paradigm.

Furthermore, in clinical validation phase, although lower dose to organs at risk in auto-plans was observed compared to those manual plans, these plans were not performed using matched comparison group designs and could be attributable to imbalances between the two cohorts. Differences in patient characteristics between the manual and auto cohorts, such as slightly lower average age and higher proportion of female patients in the auto-planned group, may have influenced dosimetric outcomes, though the improvements observed were consistent across nearly all OARs. Additionally, the goal of our study of the auto-planning system is to create acceptable plans for a majority of head and neck cases to potentially allow for the reallocation of human resources, rather than support the superiority of one method over the other. As such, we caution against interpreting the results from this study as a direct comparison between the two cohorts and continue to emphasize the importance of planner checks to correct for issues in the auto-plan. This was a single-institution experience, and generalizability to other clinical settings, planning systems, or contouring practices requires further validation. Finally, there are shortcomings to the script including a limited ability to account for specific situations such as prior delivered radiation courses, and further steps including the use of large language models to feed clinical data to augment optimization in appropriate situations (22).

Auto-planning can be leveraged to maximize efficiency, improve plan quality and minimize disparities to in low- and middle-income countries by increasing access to high-quality, and high-complexity treatment plans. Our results show that it is feasible to generate complex head and neck VMAT plans that are acceptable deemed by specialized head and neck radiation oncologists for a majority of patients. We will continue to optimize the auto-planning script to further improve plan quality, and ongoing implementation of auto-planning in large, clinical trials will be necessary to validate these findings (23).

## Conclusion

This study demonstrates that in-house automated VMAT planning for HN cancer can reproducibly generate high-quality treatment plans with superior organ-at-risk sparing, high physician acceptability, and improved efficiency compared to manual planning. Validation testing and multi-year clinical implementation in 1000 patients confirmed consistent dosimetric advantages across critical structures without compromising target coverage or dose homogeneity. Importantly, the automated workflow produced plans in under an hour with multiple optimization cycles, streamlining a historically labor-intensive process while maintaining physician confidence in clinical use. These findings highlight the feasibility and robustness of auto-planning as a scalable solution to enhance plan quality, reduce variability, and optimize resource utilization. Broader implementation of such systems has the potential to expand access to advanced head and neck radiotherapy planning, reduce disparities in care delivery, and support the integration of automation into routine clinical practice.

## Data Availability

The original contributions presented in the study are included in the article/supplementary material. Further inquiries can be directed to the corresponding author.
